# Preparation of Green Silver Nanoparticles and Eco-Friendly Polymer–AgNPs Nanocomposites: A Study of Toxic Properties across Multiple Organisms

**DOI:** 10.3390/polym16131865

**Published:** 2024-06-29

**Authors:** Lívia Mačák, Oksana Velgosova, Erika Múdra, Marek Vojtko, Silvia Dolinská, František Kromka

**Affiliations:** 1 Institute of Materials and Quality Engineering, Faculty of Materials Metallurgy and Recycling, Technical University of Kosice, Letná 9/A, 042 00 Košice, Slovakia; oksana.velgosova@tuke.sk; 2 Division of Ceramic and Non-Metallic Systems, Institute of Materials Research, Slovak Academy of Sciences, Watsonova 47, 040 01 Košice, Slovakia; emudra@saske.sk (E.M.); mvojtko@saske.sk (M.V.); 3 Institute of Geotechnics, Slovak Academy of Sciences, Watsonova 45, 040 01 Košice, Slovakia; sdolinska@saske.sk; 4 Metal Materials Division, Institute of Materials Research, Slovak Academy of Sciences, Watsonova 45, 040 01 Košice, Slovakia; fkromka@saske.sk

**Keywords:** silver nanoparticles (AgNPs), nanofibers, green synthesis, size distribution, antibacterial activity, toxicity

## Abstract

This article focuses on the eco-friendly (green) synthesis of silver nanoparticles (AgNPs) and their incorporation into a polymer matrix. For AgNPs synthesis, Lavandula angustifolia (lavender) leaf extract was used as a reducing and stabilizing agent, and as a silver precursor, AgNO_3_ solution with different concentrations of silver (50, 100, 250, and 500 mg/L) was used. Prepared AgNPs colloids were characterized using UV–vis spectrophotometry, transmission electron microscopy (TEM), and X-ray diffraction (XRD). The spherical morphology of AgNPs with an average size of 20 nm was confirmed across all samples. Further, the antimicrobial properties of the AgNPs were evaluated using the disk diffusion method on algae (*Chlorella kessleri*) and the well diffusion method on bacteria (*Staphylococcus chromogenes*, *Staphylococcus aureus*, and *Streptococcus uberis*), along with root growth inhibition tests on white mustard (*Sinapis alba*). Polymer composite (PVA–AgNPs) was prepared by incorporation of AgNPs into the polymer matrix. Subsequently, non-woven textiles and thin foils were prepared. The distribution of AgNPs within the nanocomposites was observed by scanning electron microscopy (SEM). Antibacterial properties of PVA–AgNPs composites were analyzed on bacteria *Streptococcus uberis*. It was found that not only AgNPs showed good antimicrobial properties, but toxic properties were also transferred to the PVA–AgNPs nanocomposite.

## 1. Introduction

Nanotechnology has revolutionized various industries by offering innovative solutions to complex challenges [1]. This field of science has introduced new materials at the atomic level, which possess unique properties. One of the most promising advancements in this field is the synthesis and application of nanoparticles [2,3], particularly silver nanoparticles (AgNPs). With their unique physicochemical properties [4,5], AgNPs have found widespread use in diverse fields, including medicine, electronics, catalysis, and environmental remediation [6,7]. There are several methods for producing nanoparticles. Solution chemistry using chemical and biological synthesis represents a simple production method. Green synthesis uses natural reducing and stabilizing agents such as plant extracts, which minimizes the use of toxic chemicals and reduces the negative impact on the environment. This process often takes place under normal conditions (temperature and pressure), which simplifies production and reduces costs [8]. Silver nanoparticles exhibit remarkable antibacterial properties due to their high surface-area-to-volume ratio, enabling efficient interaction with bacterial cells [9].

In recent years, there has been growing concern about the emergence of antibiotic-resistant bacteria, which pose a significant threat to public health worldwide [10]. Traditional antibiotics are becoming increasingly ineffective as bacteria develop resistance mechanisms to resist their effects. This alarming trend has prompted research into alternative antimicrobial agents, and silver nanoparticles have emerged as a promising solution [11].

Unlike conventional antibiotics, which target specific cellular components, silver nanoparticles exhibit broad-spectrum antimicrobial activity by disrupting multiple cellular processes, including cell wall synthesis, membrane integrity, and enzymatic activity [12,13,14,15,16]. Furthermore, their small size allows for enhanced penetration into bacterial cells, making them effective against Gram-positive and Gram-negative bacteria, algae, and fungi.

Moreover, silver nanoparticles have demonstrated antiviral and antifungal properties, expanding their potential applications in combating various pathogens [17]. Additionally, AgNPs exhibit excellent biocompatibility and low cytotoxicity towards mammalian cells at appropriate concentrations, making them suitable candidates for biomedical applications such as wound dressings, implant coatings, and drug delivery systems. Nanoparticles in combination with gold can also be used as a chemosensor; for example, Rohani Bastami et al. in their work used core-shell Au@Ag nanoparticles for the detection of morphine [18]. 

The incorporation of silver nanoparticles into a polymer matrix adds new properties to polymer materials, such as antibacterial [19], UV protection [20], mechanical strength [21], and electrical conductivity [22]. There are many polymers such as PVC [23], polypropylene (PP) [24], or polyvinyl alcohol (PVA) that are currently used. Polyvinyl alcohol ([CH_2_CH(OH)]_n_) is a widely used polymer; it is a linear, colorless, synthetic polymer, with a carbon backbone with hydroxyl functional groups. These hydroxyl groups can form hydrogen bonds with water and other functional groups, rendering them water-soluble and adhesive [25,26]. PVA is used for its biocompatibility and low toxicity, which means it is also environmentally friendly. For these properties, it is mainly used in medicine and the food industry. In medicine, it is used, for example, in the production of cartilage, vascular stents, and contact lenses [27]. In the food industry, it is used to produce packaging and food storage containers [28]. By incorporating silver nanoparticles into the polymer, the rare positive effects of both materials are combined. Such modern composite materials can provide not only antibacterial protection but also improve mechanical properties and extend the shelf life of food [29]. In recent years, while numerous studies have focused on the synthesis of silver nanoparticles (AgNPs) using various methods, our work stands out by employing a green synthesis approach using lavender (*Lavandula angustifolia*) leaf extract. This method not only minimizes the use of toxic chemicals, thereby reducing environmental impact, but also operates under ambient conditions, simplifying production and cutting costs. Furthermore, our study addresses a significant gap in the current literature by exploring the incorporation of AgNPs into polyvinyl alcohol (PVA) matrices to create nanocomposites with enhanced antibacterial properties.

The aim of this work is to produce silver nanoparticles and polymer nanocomposites. In the first step, using green synthesis, AgNPs will be produced. The antimicrobial properties of AgNPs will be tested on three organisms. In the second step, PMCs (polymer matrix composites) will be produced in the form of non-woven fabric and thin foil. Antibacterial properties will be also observed in the prepared polymer nanocomposites. 

## 2. Materials and Methods

Silver nitrate (AgNO_3_, >98%) purchased from Mikrochem Ltd., Pezinok, Slovakia, was used as a silver precursor. Lavender leaves were collected in a local garden in Technical University area (Košice, Slovakia) during July. Deionized water was used for solution preparation, dilution, and washing. Polyvinyl alcohol (PVA) (98.0–98.8% hydrolyzed, M.W. of approx. 146.000–186.000, purchased from Mikrochem Ltd., Pezinok, Slovakia) was used as a matrix to produce a polymer matrix nanocomposite.

The following materials were purchased for the toxicity tests: algae and bacteria were purchased from the Botanical Institute of the Academy of Sciences of the Czech Republic (Průhonice, Czech Republic), and white mustard seeds were purchased from a local gardening store. (Strain numbers: *Chlorella kessleri* CCALA 253; *Staphylococcus chromogenes*, CCM 3386 Reference No. 4497; *Staphylococcus aureus*, CCM 4750; *Streptococcus uberis*, CCM 4617.)

### 2.1. Preparation of Extract, Colloidal Solution, and Nanocomposite

The procedure for preparing lavender leaf extract was as follows: The leaves of lavender were thoroughly washed in deionized water and dried at room temperature. Subsequently, the dried leaves (5.48 g) were mixed with water and heated in a water bath at 80 °C. After heating, the extract was cooled and filtered to remove solid parts. To achieve better purity of the extract, it was centrifuged at 9000 rpm for 30 min. In this manner, 200 mL of extract was prepared.

The prepared extract was used as a reducing agent. To four flasks, 50 mL of AgNO_3_ solution with concentrations of silver 50, 100, 250, and 500 mg/L was added. Subsequently, 10 mL of extract was added to each flask with constant stirring at 600 rpm. The solution temperature was kept at 80 °C. After the addition of the entire extract, the solutions changed color from light yellow to dark brown with increasing concentrations of AgNO_3_. The color change is the first indication that biosynthesis has occurred. Colloidal solutions were subjected to UV–vis analysis after cooling to confirm the presence of silver nanoparticles. Prepared colloids served as a secondary phase for PMC matrices.

From each sample, 50 mL of colloidal solution was transferred to four plastic trays and placed in an ultrasonic bath to prevent potential nanoparticle agglomeration. Then, the samples were set to 600 rpm and heated to 80 °C. Simultaneously, PVA was added to produce 6% polymer solutions with silver nanoparticles. The composite solutions were mixed under these conditions for 45 min until the PVA powder was completely dissolved. To ensure uniform distribution of AgNPs, the composite solutions were subjected to ultrasonication for 5 min. Subsequently, two types of nanocomposites were produced—fibers and foil. 

### 2.2. Characterization of Extract, AgNPs, and Nanocomposite

Infrared (IR) spectroscopy was used to characterize the composition of the extract. The Fourier-transform infrared (FTIR) spectra were recorded using a Bruker Tensor 27 (Bruker, Billerica, MA, USA) spectrometer equipped with a GTS/KBr detector in the mid-IR region (4000–400 cm^−1^). Spectra were acquired using the KBr pellet technique (64 scans, resolution 4 cm^−1^, with a loading of 200 mg KBr and 1 mg sample, tablet diameter 13 mm). Additionally, some samples were analyzed using the attenuated total reflection (ATR) technique (64 scans, resolution 4 cm^−1^). Extract and colloid samples were dried at 45 °C in a circulating air dryer. The dried samples were mixed with potassium bromide (KBr) in a mortar and pressed into a pellet. This sample preparation procedure was consistently applied to lavender extract and colloid.

The prepared colloidal solutions of AgNPs were analyzed using a UV–vis spectrophotometer (UNICAM UV–vis Spectrophotometer UV4, Thermo Fisher Scientific Inc. Madison, WI, USA). Samples were measured in the wavelength range of 350–750 nm. The results obtained from the UV–vis spectrophotometer can provide insights into the shapes of nanoparticles, their relative concentrations, approximate sizes, and size distribution.

The size and morphology of nanoparticles were analyzed using TEM (JEOL model JEM-2000FX, with an accelerating voltage of 200 kV, JEOL Company (USA) Inc., Peabody, MA, USA,) and SEM/FIB (SEM/FIB ZEISS-AURIGA Compact, Jena, Germany). SEM analysis was used to visualize the distribution of nanoparticles incorporated into the polymer thin foil and fibers. For the AgNPs size distribution analysis, ImageJ 1.53e (ImageJ software, the National Institutes of Health and the Laboratory for Optical and Computational Instrumentation (LOCI, University of Wisconsin, Madison, WI, USA)) software was utilized.

The crystalline structure and phase composition of the samples were analyzed by X-ray diffraction (XRD, Malvern Panalytical, Almelo, The Netherlands). A laboratory X-ray diffractometer Philips X’Pert Pro in Bragg–Brentano geometry was utilized. The X-ray radiation source was a copper lamp with wavelengths *Kα*1 = 1.54056 Å and *Kα*2 = 1.54439 Å. The measurement was conducted in the range of 2Θ 20° to 100° using a rapid RTMS (real-time multiple strip) X‘Celerator detector.

Electrospinning technology was used for the preparation of non-woven textiles. The voltage used in the electrospinning process was 72 kV (Liberec, Czech Republic), and the distance between the rotating and receiving electrodes was 120 mm. Foil was produced by casting thin layers on a non-adhesive substrate.

### 2.3. Antimicrobial Activity of AgNPs

To test the antimicrobial properties, three different types of already-identified microorganisms were chosen: algae, bacteria, and plants. 

The first test was realized on the green algae *Chlorella kessleri* (*Ch. kessleri*), using the standard disk diffusion method. On prepared Milieu Bristol agar plates in Petri dishes, the green alga *Ch. kessleri* was inoculated. For each tested sample, 15 µL of AgNPs colloids was dropped onto sterile disks with a diameter of 5 mm. Subsequently, the agar plates were incubated at room temperature under light/dark conditions for 12:12 h. In addition, the extract was also tested as a negative control and AgNO_3_ (500 mg/L) as a positive control. The inhibition zone was recorded after 10 days of incubation. This method measured the ability of the tested samples and controlled substances to inhibit the growth of microorganisms.

In the second test, the antibacterial properties of AgNPs and their effectiveness against selected pathogens, *Staphylococcus chromogenes* (*S. chromogenes*), *Staphylococcus aureus* (*S. aureus*), and *Streptococcus uberis* (*S. uberis*), were monitored. The antimicrobial activity was tested in four different concentrations of AgNPs to determine the optimal concentration for antimicrobial activity. The test was realized using the well diffusion method with a well diameter of 2 mm. The results of the toxicity tests were faster than the algal tests, and inhibition zones could be recorded after 24 h.

The third method was a seed and root growth inhibition test on a higher plant. The standard for testing was STN 83 8303 ecotoxicity, acute toxicity tests on higher cultivated plants. White mustard seeds were used to test AgNPs colloids with concentrations of 50, 100, 250, and 500 mg/L. The procedure for individual concentrations was as follows: the seeds were sterilized (30 pieces for one concentration), the seeds were immersed for 20 min into a 10% solution of sodium hypochlorite (NaClO), and then they were washed with distilled water (around 300 mL) until the presence of chlorine was no longer felt. Filter papers in each Petri dish were placed, and 10 mL of the colloidal solutions was poured. Then, sterilized white mustard seeds were evenly placed in each Petri dish in a 6 × 5 rectangular arrangement. The prepared Petri dishes were stored in a dark room at room temperature and monitored for three days. Germination and root growth were evaluated. With this toxicity test, we evaluated how many seeds germinated (the dependence of the number of days on the percentage of germinated seeds) and the length of the roots of the germinated seeds.

### 2.4. Antibacterial Activity of Nanocomposites

Nanocomposite fibers and foil containing AgNPs were tested on an agar plate with inoculated *S. uberis* bacteria. Disks with a diameter of 5 mm were cut from individual nanocomposites and placed on an inoculated agar. The inhibition zones were evaluated after 24 h.

## 3. Results and Discussion

### 3.1. Characterization of Lavender Extract and Nanostructures

**Figure 1:**
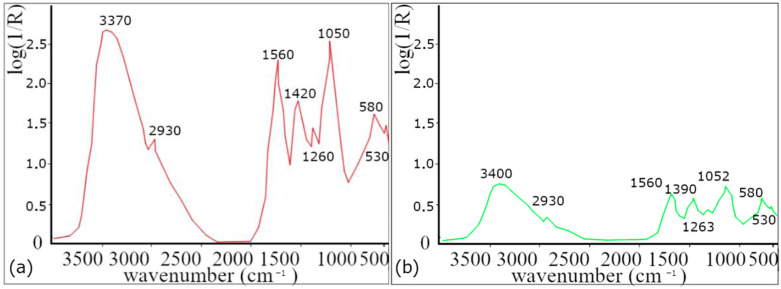
FTIR analysis. (**a**) Extract from dried lavender leaves and (**b**) colloidal solution for a concentration of 50 mg/L.

Using FTIR analysis, the functional groups contained in the lavender leaf extract were identified Figure 1. 

Absorption bands at 2860 and 2930 cm^−1^ are attributed to valence vibration of −CH_2_ groups, while the band at 3400 cm^−1^ corresponds to valence vibrations of O−H groups [30,31,32], which indicates the presence of alcohol (e.g., linalool and terpene−4−ol) [33]. The absorption band at 1740 cm^−1^ is assigned to the valence vibrations of the C=O group, indicating the presence of carbonyl groups of esters such as linalyl acetate, camphor, and Lavandula acetate [33], and the band at 1640 cm^−1^ belonging to the valence vibration of the C=C group is also present in both samples [31,32,33,34] and found in components such as linalool, linalyl acetate, terpene−4−ol, and β−caryophyllene [33]. The presence of other ester components is also confirmed by the band at 1240 cm^−1^, corresponding to the C−O valence vibration of the ester group [32]. The band around 1420 cm^−1^ corresponds to the C-O-H group. These functional groups are contained in proteins and flavonoids. 

Based on the decrease in the spectra of the AgNPs colloid, it is possible to determine which functional groups, and thus also the compounds containing these functional groups, were involved in the synthesis of nanoparticles. Due to the complex composition of lavender, it is difficult to identify which functional groups were involved in the reduction process of silver ions and which were responsible for the stabilization of AgNPs. According to our results (change/decrease in band intensity) and according to the available literature, proteins and flavonoids are mostly responsible for the synthesis.

**Figure 2:**
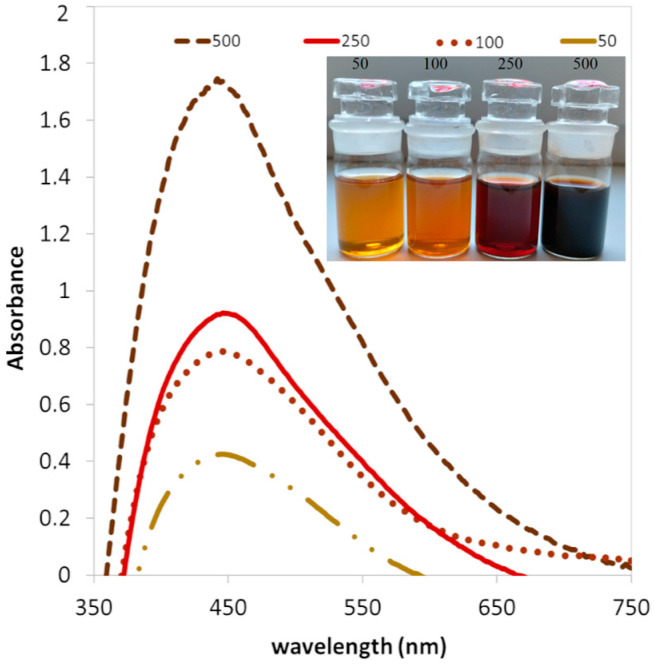
UV–vis absorbance of AgNPs colloids with different concentrations of AgNO_3_ (50, 100, 250, and 500 mg/L).

UV–vis analysis confirmed the presence of nanoparticles in all prepared colloidal solutions. The analysis results (Figure 2) clearly showed the characteristic absorption peaks for spherical silver nanoparticles in the range of 442−446 nm for each sample [35,36,37]. These results confirm the successful synthesis of silver nanoparticles [38].

It is well known that silver nanoparticles in aqueous solutions exhibit a change in color intensity depending on their concentration and size [39]. UV–vis absorption spectra were measured at different concentrations of the precursor (50, 100, 250, and 500 mg/L) at a constant volume of extract (10 mL). Increasing absorbance intensity was observed with increasing precursor concentration. The color change for individual samples was also confirmed, see the inner picture in Figure 2; with a higher concentration, a more intense color can be observed. The redox reaction in AgNPs synthesis is based on the reduction of silver ions (Ag^+^) to elemental silver (Ag^0^) using a reducing agent. In the case of biological synthesis, the reducing agents are often biomolecules such as proteins, enzymes, or phytochemicals present in plant extracts. The color change is a direct visual indicator of the formation of nanoparticles, which confirms the reduction of silver ions. During the formation of AgNPs, the color of the initial solution changes because the nanoparticles exhibit surface plasmon resonance (SPR) [40]. The principle is that their electrons resonate with the wavelengths of light, and this subsequently leads to a change in the color of the colloid. It is obvious that the lavender leaf extract is a successful agent with a high capacity to reduce Ag^+^ ions to Ag^0^.

**Figure 3:**
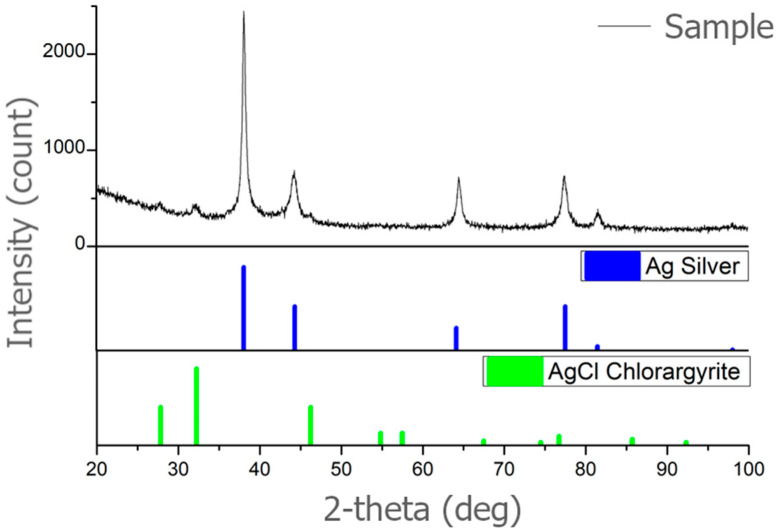
XRD analysis of AgNPs synthesized with lavender leaf extract (sample 500 mg/L).

Using XRD, the crystallographic phases in the sample (500 mg/L) were identified. Comparing the XRD spectrum of samples with the PDF−2 database (powder diffraction file), the silver was confirmed; silver shows characteristic peaks at 2° values of 38.10°, 44.37°, 64.18°, and 77.55°, see Figure 3. These peaks are assigned to the (111), (200), (220), and (311) planes, which are characteristic planes for the crystal structure of silver PDF Ag 00-001-1167. The content of the silver was 91.6%. The presence of AgCl PDF 00-031-1238 was also detected in the representation of 8.4%, see Table 1.

It was confirmed that the analyzed sample is silver (Ag). Confirmation of a cubic lattice specifies that the crystal structure of the silver nanoparticles is cubic, with a unit cell that is a cube with equal edge lengths and angles of 90 degrees. Space group Fm-3m is the specific space group notation for this cubic lattice structure. “Fm-3m” is a notation used in crystallography to describe the crystal lattice’s symmetry. It belongs to the face-centered cubic (fcc) crystal system and indicates that the lattice has a high degree of symmetry, including mirror planes and rotational axes. This analysis confirms it as a common and stable form for metallic silver.

### 3.2. TEM Analysis of AgNPs

**Figure 4:**
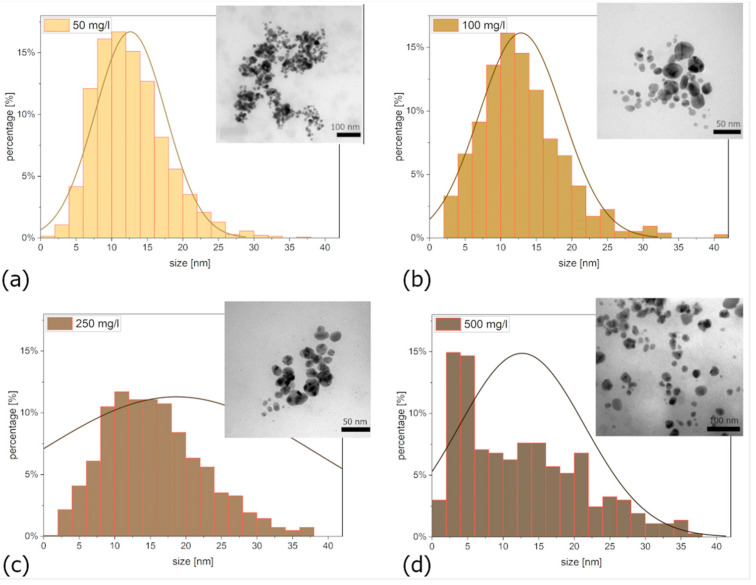
Size distribution of nanoparticles (**a**) 50 mg/L, (**b**) 100 mg/L, (**c**) 250 mg/L, and (**d**) 500 mg/L; the inner pictures show the TEM nanostructure of AgNPs.

Figure 4a–d show the histograms of the nanoparticle size distribution in the samples prepared using precursors with different concentrations of Ag^+^ ions. In the case of a sample with a concentration of 50 mg/L, we found that nanoparticles with a size of up to 20 nm represented 91.91%.

In the case of a sample with a concentration of 100 mg/L, this value was 89.28%. For a sample with a concentration of 250 mg/L, nanoparticles with a size up to 20 nm made up 73.24%, and for a sample with a concentration of 500 mg/L, it was 78.39%. TEM analysis confirmed that all colloidal solutions contained nanoparticles of spherical shape, see the inner pictures in Figure 4.

It is generally understood that for achieving good toxicological properties, smaller nanoparticles are most advantageous. However, for other applications, larger nanoparticles may be more suitable. The kinetics of the synthesis process also play a crucial role. Faster synthesis results in smaller nanoparticles. This is evident in Figure 4a, where an excess of extract led to the formation of numerous small nanoparticles. Moreover, increasing the silver precursor concentration expands the distribution towards larger sizes. From a synthesis efficiency perspective, higher precursor concentrations are preferred. However, considering toxicity, producing a large number of smaller nanoparticles would be more desirable.

### 3.3. Polymer Matrix Nanocomposites

**Figure 5:**
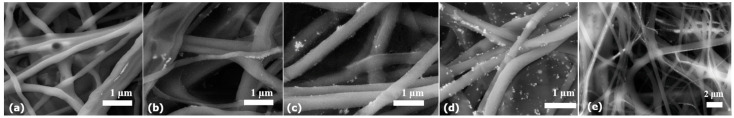
PVA–AgNPs nanocomposite fibers prepared by AgNPs colloids of different concentrations: (**a**) 50 mg/L, (**b**) 100 mg/L, (**c**) 250 mg/L, (**d**) 500 mg/L (nanoparticles on the surface), and (**e**) 500 mg/L (AgNPs inside the fibers).

**Figure 6:**
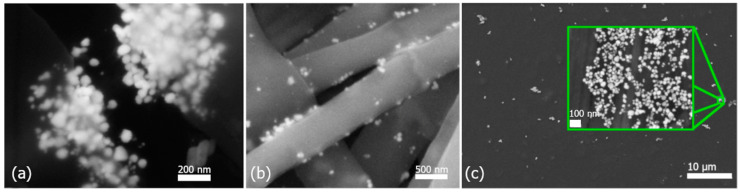
Incorporated AgNPs at 500 mg/L in nanofibers (**a**) inside a fiber, (**b**) on the surface of fibers, and (**c**) in thin foil.

PVA polymer was used as a matrix, and silver nanoparticles prepared by a biological method (in concentrations: 50, 100, 250, and 500 mg/L) were used as the secondary phase. The fiber nanocomposites were analyzed using scanning electron microscopy for different concentrations of incorporated colloidal solutions (Figure 5). It is clear that colloids with a lower concentration (50 and 100 mg/L) contain a sufficient amount of nanoparticles, and therefore, nanoparticles are not present in all fibers (Figure 5a,b). On the other hand, at higher concentrations of AgNPs (Figure 5c–e), it is clear that the fibers contain significantly more nanoparticles; nanoparticles were present in 90% of the fibers. Despite the higher representation of nanoparticles in the fibers, it is possible to observe an uneven distribution of nanoparticles. Nanoparticles are present on the surface of some fibers (Figure 5d) and in their interior (Figure 5e), but in detailed view, clusters of nanoparticles can also be observed (Figure 6a).

Clusters could be observed mainly in the fibers of samples with a higher concentration of AgNPs (500 mg/L). Therefore, these samples were subjected to a more detailed study. Figure 6a shows a fiber with a cluster of nanoparticles after tearing the fiber. The nanoparticles are encapsulated inside the fiber; at the same time, it is clear that the polymer covers the entire cluster and not individual nanoparticles. Such clusters, in certain applications, may also be advantageous, for example, in cases where a burst release of a larger amount of an antibacterial component is required. The cluster shell initially protects the nanoparticles, and after tearing/dissolving, the nanoparticles can directly react with the environment. To achieve better results in the distribution of nanoparticles, alternative methods of incorporation should be considered. The in situ method, the use of more intense mixing, or ultrasound in homogenization after the nanoparticle’s incorporation into the matrix should be considered.

In Figure 6b, the nanoparticles that are on the surface of the fiber can be seen in detail. Since the nanoparticles are not covered by a polymer layer, it is possible to assume a faster toxic effect than in the case of nanoparticles incorporated inside the fiber. Nanoparticles on the fibers’ surface are entrapped in the pores of the nanofibers. Shetty et al. in their work confirmed that cation–anion interactions, i.e., electrostatic attraction, are the basis for keeping nanoparticles on the fiber surface [41].

In addition to the fibers, a thin foil was prepared (Figure 6c). It is clear that the nanoparticles are not distributed uniformly but form clusters (inner picture of Figure 6c). The nanoparticles in the cluster are spherical, and each nanoparticle is surrounded by polymer. Nanoparticles do not form encapsulated clusters as in the case of Figure 6a. By more intensive homogenization, it would probably be possible to achieve a more uniform distribution of nanoparticles throughout the entire volume of the thin foil.

### 3.4. Toxicity of AgNPs Colloids and PVA–AgNPs Nanocomposites

**Figure 7:**
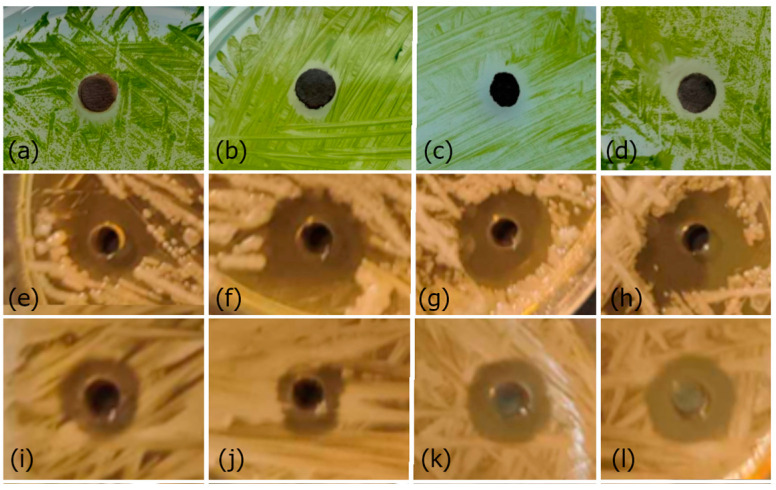
Antibacterial test of AgNPs’ effect against *Ch. kessleri* for samples: (**a**) 50 mg/L, (**b**) 100 mg/L, (**c**) 250 mg/L, (**d**) 500 mg/L; *S. chromogenes:* (**e**) 50 mg/L, (**f**) 100 mg/L, (**g**) 250 mg/L, (**h**) 500 mg/L; and *S. aureus*: (**i**) 50 mg/L, (**j**) 100 mg/L, (**k**) 250 mg/L, (**l**) 500 mg/L. Disks with a diameter of 5 mm were used for samples (**a**–**d**), and wells with a diameter of 2 mm were used for samples (**e**–**l**).

A toxicity test was performed to determine the resistance level of algae (Figure 7a–d) and bacteria (Figure 7e–l) to AgNPs. After the incubation of individual organisms, clear inhibition zones were observed. It is obvious that as the concentration of individual samples increases, so does the inhibition zone. The antibacterial test was repeated three times for each sample, and the averaged measurement results are shown in Table 2.

The results confirmed that in all tested organisms, the inhibition zone increases with the increasing concentration of AgNPs. In the case of testing bacteria, it is clear that the largest inhibition zones were measured in the case of *S. chromogenes* bacteria. It should be noted that nanoparticles have a more significant effect on bacteria compared to algae. Although a different type of test was used in the case of algae testing (when compared to the size of the disk (5 mm) or well (2 mm)), it is clear that more significant inhibition zones were created in the case of bacteria.

**Figure 8:**
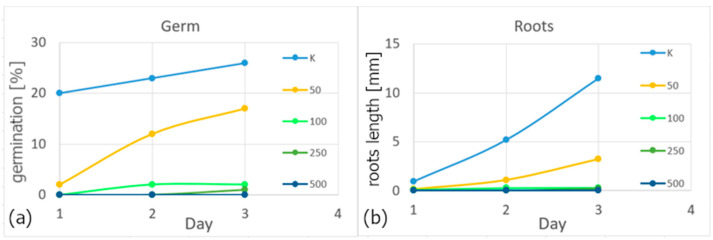
Evaluation of germination (**a**) and root growth (**b**) of white mustard.

Based on their different concentrations, the properties of colloids were also tested on white mustard seeds using the inhibition test on a higher plant. Germination and root growth were evaluated. It is clear from the results that the toxic effect also increases with increasing concentration, see Figure 8. Water was used as a control (K). In both cases, it is clear that the 500 mg/L colloidal solution has a 100% toxic effect; within three days, no growth occurred in the case of seeds or roots. 

**Figure 9:**
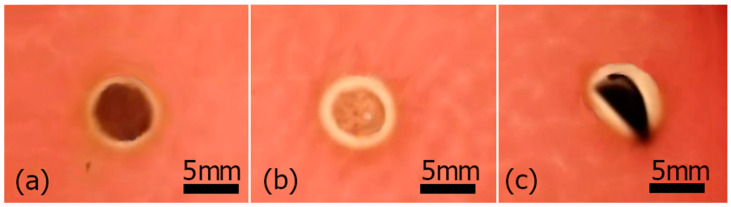
Antibacterial activity of colloid 500 mg/L (**a**), non-woven fabric (**b**), and foil (**c**).

The antibacterial properties of colloid 500 mg/L, PVA–AgNPs nanocomposites non-woven fabric, and foil were tested on an agar plate inoculated with a strain of *S. uberis* bacteria. AgNPs colloidal solution (6.68 mm), fibers (7.12 mm), and foil (7.22 mm) showed distinct inhibition zones (Figure 9). The foil in Figure 9c has a different shape; rolling is caused by thickness and humidity.

The inhibitory activity of AgNPs against bacteria and other organisms can occur through different mechanisms. AgNPs can damage the cell mechanically, enter the cell, and affect intracellular structures (mitochondria, vacuoles, ribosomes, and DNA), which can cause damage to the cell or prevent its reproduction. AgNPs can induce cell toxicity by producing reactive oxygen species (ROS), which affects cell signaling and subsequently leads to cell death [42,43]. Reactive forms of oxygen (ROS—reactive oxygen species), so-called free radicals, are chemical compounds containing oxygen that have unstable electrons and can react with other substances in the cell. Reactive oxygen forms are also created in the cell during its normal functioning process. However, AgNPs are catalytic and increase the amount of ROS, and, as a result, oxidative stress (OS) will occur, which leads to the violation of its functions and damage.

The toxic effect of nanoparticles is strongly dependent on the shape, size, stability, and concentration of the nanoparticles [44,45]. Based on the results, it is clear that for the same shape and size of nanoparticles, the concentration of nanoparticles plays a key role. 

Ali et al. [46], in their work, prepared AgNPs by green synthesis using an extract from the leaves of Eucalyptus globulus. Using the disk diffusion test, they tested antibacterial effects on Gram-positive and Gram-negative bacteria. The results of the tests showed that with increasing concentrations of AgNPs, the inhibition zones also increased. Antibacterial effects were confirmed for all colloidal silver solutions. 

In a detailed analysis of seed toxicity on higher plants, it was demonstrated that colloidal silver solutions can affect seed germination and the growth of small plants. Such toxicity tests represent an interesting contribution in the area of the impact of nanoparticles on the environment. 

This influence is particularly notable in the early stages of seed growth, where the cell count is lower compared to larger plants. These findings suggest a potential for the use of colloidal silver in applications such as plant sprays. Colloidal silver could be effective in controlling pests without damaging the entire plant. While other studies focus on the chemical synthesis of silver nanoparticles, our biologically synthesized colloids exhibit lower toxicity to plants while maintaining high antibacterial efficacy. Moreover, the biological synthesis of colloidal silver is an effective and environmentally friendly alternative to traditional chemical methods. Colloidal silver can be used as a final product, and incorporating colloidal solution into matrices can expand its applications. The results showed that not only do AgNPs have good antibacterial properties, but they can also transfer these properties to the polymer matrix. A synergistic effect on antibacterial activity with incorporated AgNPs in PMC was achieved. The fibers and foils exhibited similar antibacterial properties to those reported by Gudimalla et al. [47] in their work. Although they used a different polymer (sodium alginate), they confirmed that nanoparticles not only enriched the matrix with antibacterial properties but also increased the strength of the resulting composite.

Polymers are easy to process, which enables the production of materials of various shapes and sizes, adapted to specific applications. The created polymer matrix thin foil enriched with AgNPs appears to be a promising material. It has potential applications especially in the food industry for food packaging to ensure longer freshness and protection against bacterial contamination. In addition, nanocomposites in the form of fibers represent an interesting alternative in medicine, for example, in the creation of artificial skin for the treatment of burns, where the AgNPs content contributes to the promotion of healing and the prevention of inflammatory and infectious reactions.

The use of ecological methods of green synthesis contributes to sustainable development and environmental protection.

## 4. Conclusions

Colloidal solutions of AgNPs with different concentrations of silver (50, 100, 250, and 500 mg/L) were successfully prepared using green synthesis. An extract from dried lavender leaves was used as a reducing and stabilizing agent. FTIR analysis showed that proteins and flavonoids contained in the extract are mostly responsible for the synthesis of AgNPs. UV–vis and TEM analyses confirmed the presence of spherical silver nanoparticles with an average diameter of ~20 nm in all samples. The toxicity of the prepared silver nanoparticles was tested and proved on three different organisms (algae, bacteria, and plants). The results indicate that the colloidal solution at a concentration of 500 mg/L demonstrates the highest efficacy across all toxicity tests.

The prepared silver nanoparticles (all concentrations) were incorporated into a 6% PVA solution by an ex situ method. Such a polymer composite (PVA–AgNPs) was used to prepare non-woven fabrics and thin foil. The distribution of AgNPs depending on the concentration used was observed using SEM analysis. The highest concentrations showed better distribution. However, even at higher concentrations, the distribution was not uniform, especially for the foil. 

## Figures and Tables

**Table 1:** Analysis of AgNPs (concentration 500 mg/L) prepared by lavender leaf extract.

Sample	Lattice	Space Group	Vol. Percentage (%)
Ag	Cubic	Fm-3m	91.6
AgCl	Cubic	Fm-3m	8.4

**Table 2:** The size of the inhibition zones of different concentrations of AgNPs for algae and individual strains of bacteria.

Sample	*Ch. kessleri*	*S. chromogenes*	*S. aureus*
50 mg/L	6.98	5.54	5.20
100 mg/L	7.62	6.16	5.80
250 mg/L	8.72	6.71	5.80
500 mg/L	9.09	7.09	5.91

## Data Availability

The original contributions presented in the study are included in the article; further inquiries can be directed to the corresponding author.
